# Characterization of the complete mitochondrial genome of *Ypthima baldus* (Lepidoptera: Satyrinae) with phylogenetic analysis

**DOI:** 10.1080/23802359.2020.1721348

**Published:** 2020-02-03

**Authors:** Xiao-Dong Li, Hao-Wu Hu, Shu-Li Zhang, Jia-Wei Wang, Ran Li

**Affiliations:** a School of Chemistry and Bioengineering, Hechi University, Yizhou, Guangxi, P. R. China;; b The Key Laboratory of Jiangsu Biodiversity and Biotechnology, College of Life Sciences, Nanjing Normal University, Nanjing, Jiangsu, P. R. China;; c School of Plant Protection, Nanjing Agricultural University, Nanjing, Jiangsu, P. R. China

**Keywords:** Lepidoptera, *Ypthima baldus*, mitogenome, phylogenetic analysis

## Abstract

The complete mitochondrial genome of *Ypthima baldus* was determined and analysed for the first time. It is a circular molecule of 15,304 bp in length and contains 13 protein-coding genes (PCGs), 2 rRNA genes (12S and 16S), 22 tRNA genes (tRNAs), and 1 AT-rich region. The total base composition is 38.6% of A, 7.5% of G, 42.2% of T, and 11.7% of C, respectively. In addition, phylogenetic analysis was carried out with 13 PCGs using the Bayesian Inference (BI) method. The complete mitogenome of *Y. baldus* will play an important role in population genetics and phylogenetic studies of the species in the future.

Mitochondrial genome (mitogenome) of insect is usually a circular molecule spanning 14–20 kb in size (Boore 1999). Mitogenomes exhibit several properties, such as rapid evolutionary rate, small genome size, low recombination, and maternal inheritance, thus are being increasingly employed to explore the evolution and phylogenetic relationships in diverse insect taxa (Cameron 2014; Dai et al. 2018). *Ypthima baldus* is one species of genus *Ypthima*, comprising over 100 species found on all continents except Antarctica (Ackery et al. 1999). Until now, only one sequence of the mitochondrial DNA of the genus is available (Wu et al. 2014).

Here, the complete mitogenome of *Y. baldus* was revealed based on the samples collected from Yizhou in Guangxi Province, China. All samples were stored in 95% ethanol at temperature –20 °C and deposited in the Museum of Insects of Hechi University (the voucher No. L385), Yizhou, Guangxi. The total genomic DNA was extracted from thorax muscle of an adult specimen using a Wizard^®^ Genomic DNA Purification Kit (Promega, Madison, USA). Using certain pairs of universal primers (Simon et al. 2006), the mitochondrial genome was amplified by polymerase chain reaction (PCR). Then, PCR products were sequenced using primer-walking strategy from both strands. Mitochondrial sequence was assembled by SeqMan program from DNASTAR (Burland 2000) and annotated using MEGA 7.0 software (Kumar et al. 2016) with reference to the mitogenome of *Ypthima akragas* (GenBank accession KF590553).

The complete mitogenome of *Y. baldus* (GenBank accession No. MN708051) was 15,304 bp in length, containing typical insect 13 protein-coding genes (PCGs), 2 ribosomal RNA genes (rRNAs), and 22 transfer RNA genes (tRNAs), along with one AT-rich control region. Its gene arrangement and orientation were consistent with those of other known nymphalid mitogenomes (Shi et al. 2019). The overall nucleotide composition was 38.6% of A, 7.5% of G, 42.2% of T, and 11.7% of C, with the AT-rich feature (80.8%). All protein-coding sequences had a typical ATN codon with the exception of the COI which used the unusual CGA. The mitogenome contained four PCGs (COI, COII, ND5, ND4) with single-nucleotide (T) stop codons completed by adding 3′A residues post-transcriptionally. The 22 typical tRNA genes were interspersed in the genome and range from 59 to 71 bp in length. The 12S and 16S rRNA genes were 779 bp and 1358 bp in size, respectively. The A + T-rich region of the mitogenome was located in the conserved position between the 12S and tRNA^Met^ with a length of 370 bp.

To understand the phylogenetic relationship of *Y. baldus* with other Satyrinae species, a Bayesian Inference (BI) phylogenetic tree was constructed on CIPRES Portal using 13 mitochondrial PCGs from mitogenomes of 17 species (see Figure 1 for details). The phylogenetic analysis showed that *Y. baldus* formed a monophyletic group with other Satyrinae species and *Y. baldus* was positioned near *Y. akragas*, which indicated that our newly determined mitogenome sequence will be useful for studying population genetics and phylogenetic analysis of this species.

**Figure 1.**
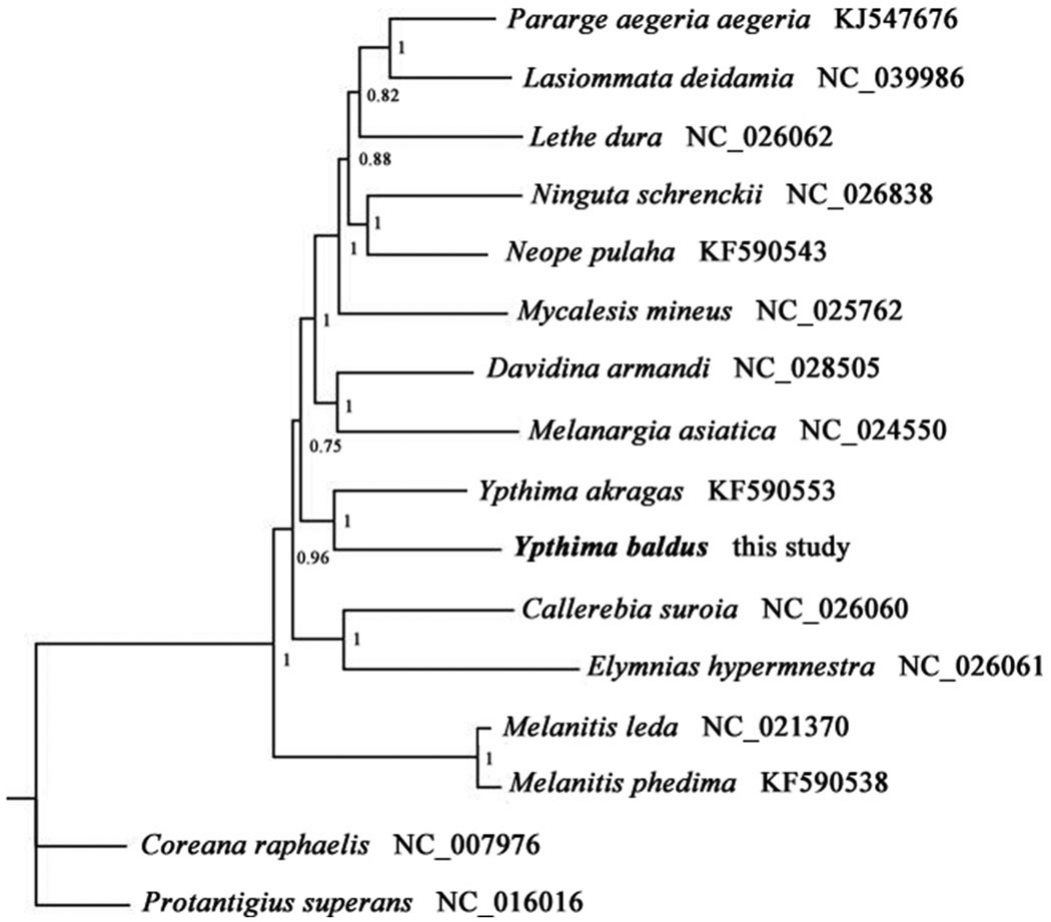
Phylogenetic tree obtained from BI analysis based on 13 concatenated mitochondrial PCGs. Numbers on node are posterior probability (PP).
